# Effect of the Substrate Crystallinity on Morphological and Magnetic Properties of Fe_70_Pd_30_ Nanoparticles Obtained by the Solid-State Dewetting

**DOI:** 10.3390/s21217420

**Published:** 2021-11-08

**Authors:** Gabriele Barrera, Federica Celegato, Matteo Cialone, Marco Coïsson, Paola Rizzi, Paola Tiberto

**Affiliations:** 1 Advanced Materials Metrology and Life Sciences, Istituto Nazionale di Ricerca Metrologica (INRiM), Strada delle Cacce 91, I-10135 Torino, Italy; f.celegato@inrim.it (F.C.); m.coisson@inrim.it (M.C.); p.tiberto@inrim.it (P.T.); 2 CNR SPIN Genova, c.so F. M. Perrone 24, I-16152 Genova, Italy; matteo.cialone@spin.cnr.it; 3 Chemistry Department and NIS, Università di Torino, Via Pietro Giuria 7, I-10125 Torino, Italy; paola.rizzi@unito.it

**Keywords:** FePd thin film, solid-state dewetting, substrate crystallinity, magnetic nanoparticles

## Abstract

Advances in nanofabrication techniques are undoubtedly needed to obtain nanostructured magnetic materials with physical and chemical properties matching the pressing and relentless technological demands of sensors. Solid-state dewetting is known to be a low-cost and “top-down” nanofabrication technique able to induce a controlled morphological transformation of a continuous thin film into an ordered nanoparticle array. Here, magnetic Fe${}_{70}$Pd${}_{30}$ thin film with 30 nm thickness is deposited by the co-sputtering technique on a monocrystalline (MgO) or amorphous (Si${}_{3}$N${}_{4}$) substrate and, subsequently, annealed to promote the dewetting process. The different substrate properties are able to tune the activation thermal energy of the dewetting process, which can be tuned by depositing on substrates with different microstructures. In this way, it is possible to tailor the final morphology of FePd nanoparticles as observed by advanced microscopy techniques (SEM and AFM). The average size and height of the nanoparticles are in the ranges 150–300 nm and 150–200 nm, respectively. Moreover, the induced spatial confinement of magnetic materials in almost-spherical nanoparticles strongly affects the magnetic properties as observed by in-plane and out-of-plane hysteresis loops. Magnetization reversal in dewetted FePd nanoparticles is mainly characterized by a rotational mechanism leading to a slower approach to saturation and smaller value of the magnetic susceptibility than the as-deposited thin film.

## 1. Introduction

Nanotechnology and sensors are research areas with a high degree of multi- and inter-disciplinarity and, therefore, they are often and continuously combined [1,2,3]. Therefore, the development of advanced sensors based on nanostructured materials is increasingly expanding, improving the detection sensitivity of several chemical, physical and biological quantities in several external conditions [4,5,6,7,8,9,10]. In this framework, nanostructures-based bio-sensors are already used to sense various signals from a wide range of biological environments with many technological advantages in terms of reducing cost, easing in technologies, and improving measurement efficiencies [8,11,12,13]

Recent advances and tuning of innovative nanofabrication techniques have allowed to finely control the size and shape of the nanostructured materials, tailoring their physical and chemical properties to the practical demands [14,15,16]. Moreover, the more suitable nanofabrication technique should take into account also the economic issues, whose aim is the cost reduction of the sensor with its diffusion in the global market [14].

In this context, solid-state dewetting is a physical, “top-down” and low-cost nanofabrication technique [17,18] able to induce a morphological transformation of a thin film into an ordered nanoparticles array, usable as a key component in catalysts, photonic and magnetic applications as well as in several sensors [19,20,21,22]. The thermally activated morphological transformation is driven by a reduction in the surface energy of the thin film and, strictly depends on the interface energy between the thin film and the underlying substrate [18]. Therefore, the final shape and size of the nanoparticles (NPs) lying on the substrate are strongly influenced by well-known factors, including the thickness and composition of the thin film, temperature, time, and atmosphere of annealing treatment, as well as the physical and chemical properties of the substrate surface [23,24].

The solid-state dewetting process applies to a wide class of materials and alloys [19,22,24,25,26,27]. Interestingly, this process occurring in noble metals (Au and Ag) supports the development of the plasmonic sensors based on surface-enhanced Raman scattering (SERS) effect by the fabrication of suitable nanostructured substrates with high performance and low cost [21,28,29]. On the other hand, the solid-state dewetting applied to single- and multi-layer magnetic thin film strengthens the understanding of the correlation between the technologically relevant magnetic properties, such as anisotropy, susceptibility, coercivity, remanence and the morphological features of the obtained NPs [24,25,30].

Using the solid-state dewetting process to obtain bimetallic NPs, one element of which is magnetic, is an interesting scientific goal. The magnetic bimetallic NPs are promising materials in the technological and research communities because they show both the superimposition of the properties of the single elements and new interesting combined effects [31,32,33,34]. Among others, the bimetallic FePd NPs, thanks to their well-known multifunctional properties [35,36], have been already used as glucose sensors [37], biomedical agents [38,39], high-efficiency (bio-)catalysts [40], SERS-active substrate [41,42], and in magnetorheological fluid [36].

The aim of this work is to propose the solid-state dewetting process as an efficient nanofabrication technique able to obtain an array of bimetallic Fe${}_{70}$Pd${}_{30}$ nanoparticles starting from a continuous thin film deposited by the sputtering technique on a substrate. This research study is mainly devoted to understanding how the microstructure of the underlying substrate affects this morphological transformation and can be used as a tool to meet technological needs. In particular, monocrystalline MgO and amorphous Si${}_{3}$N${}_{4}$ substrates are used to control the kinetics of the dewetting process and to tailor the final morphology of magnetic FePd nanoparticles. The structural and morphological properties of the as-deposited FePd thin film and subsequently the size, shape, density and distribution of the obtained FePd NPs attached to the underlying substrate are investigated by X-ray diffractometer, scanning electron microscopy (SEM) and atomic force microscopy (AFM). Moreover, the evolution of the in-plane and out-of-plane magnetic properties, directly related to the spatial confinement induced by the dewetting process, are carefully investigated.

## 2. Materials and Methods

The co-sputtering deposition technique on monocrystalline MgO or amorphous Si${}_{3}$N${}_{4}$ substrate was used to grow FePd alloy in a thin-film form with 30 nm thickness. The two substrates were simultaneously loaded into the sputtering chamber. The power density of the two targets was fixed based on their relative deposition rates, at 250 W for Fe element and 15 W for the Pd element, in order to obtain the desired Fe${}_{70}$Pd${}_{30}$ stoichiometry. The base pressure of the sputtering was set at 7 × 10${}^{-7}$ mbar and the Ar gas pressure at 1.2 × 10${}^{-2}$. The deposition time, to obtain the desired 30 nm thickness, was calculated from the deposition rate of FePd (1.36 Å/s) which was previously experimentally evaluated by an atomic force microscopy (AFM) measurement. The two as-deposited samples are named as follows: FePd/MgO and FePd/SiN.

The as-deposited thin films were submitted to annealing in a furnace (carbolite) (heating rate 51 °C/min) under vacuum atmosphere (2 × 10${}^{-6}$ mbar) to promote the solid-state-dewetting process. The selected annealing temperatures (*T*${}_{A}$) are in the range 750–860 °C, and the annealing time (*t*${}_{A}$) is set to 55 min. Despite the high temperatures reached during the annealing process, the high vacuum atmosphere in the furnace chamber severely hinders the oxidation of the FePd surface.

The crystal structure of the as-deposited FePd thin film was investigated by means of the grazing incidence X-ray diffraction (GIXRD) technique with Cu-K α radiation (PANalytical X’Pert Pro MPD). The XRD spectra were collected at room temperature. The Scherrer formula was used to estimate the average grains size [43]. The morphological characterization of the continuous as-deposited thin films and dewetted samples was performed by scanning electron microscopy (SEM—FEI Inspect-F). The corresponding images were analyzed by an open source software ImageJ [44]. The energy dispersive X-ray spectrometer (EDS) equipped in the SEM was used to check the stoichiometry of the as-deposited FePd alloy.

The surface roughness of the as-deposited samples and the height maps of the dewetted ones were measured by AFM (Bruker Multimode V Nanoscope 8) operating in intermittent-contact mode at atmospheric pressure and room temperature. In-plane and out-of-plane room temperature magnetic hysteresis loops were measured by means of a high-sensitivity alternating gradient field magnetometer (AGFM, Princeton Measurements Corporation) operating in the field range −18 ≤ H ≤ 18 kOe. The magnetic signal of the sample holder and substrate was effectively subtracted.

## 3. Results

### 3.1. As-Deposited Fe${}_{70}$Pd${}_{30}$ Thin Film

#### 3.1.1. Structural and Morphological Characterization

The Fe:Pd ratio in the alloy, experimentally evaluated by the EDS technique, is Fe${}_{70}$Pd${}_{30}$ for the thin film deposited both on the monocrystalline MgO and amorphous Si${}_{3}$N${}_{4}$ substrates.

**Figure 1:**
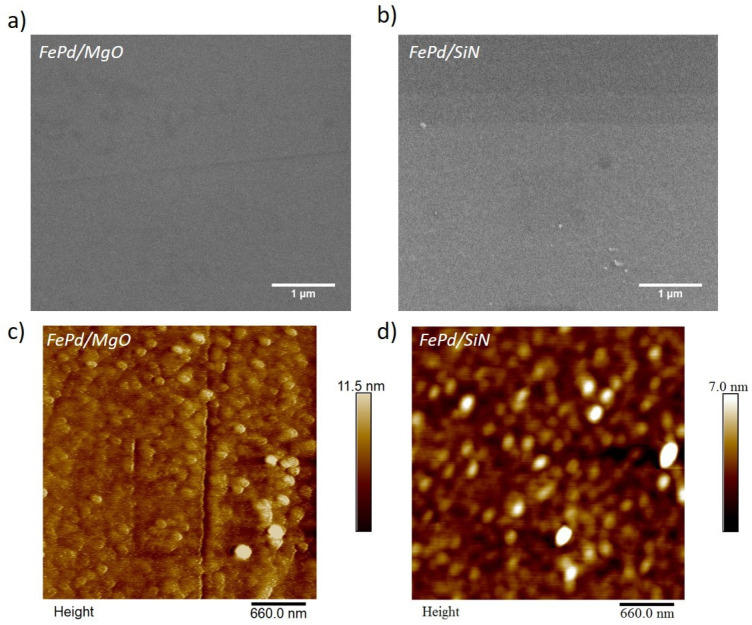
SEM (panels **a**,**b**) and AFM (panels **c**,**d**) images of as-deposited FePd thin films on MgO or Si${}_{3}$N${}_{4}$ substrate.

The surface morphology of the as-deposited FePd/MgO and FePd/SiN samples is depicted by top-view SEM and AFM images (see Figure 1). In particular, the SEM micrographs (Figure 1a,b) show a uniform and flat surface without visible defects and macro-structures in both samples. AFM images (Figure 1c,d) confirm the high surface flatness, revealing a roughness with a round shape with a root mean square (R${}_{q}$) values of 1.4 nm and 1.1 nm for FePd/MgO and FePd/SiN samples, respectively. These measured R${}_{q}$ values are too low to induce a contrast/brightness variation in the SEM images, which appear to be mainly monochrome. The absence of visible defects or cracks indicates a low accumulation of strain at the substrate/film interface during the FePd deposition for both substrates [45,46].

**Figure 2:**
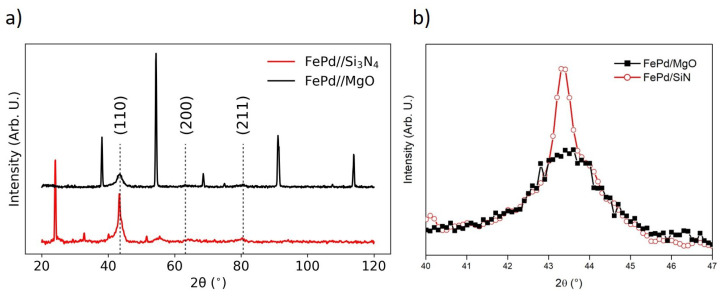
(**a**) XRD spectra of the as-deposited FePd samples on MgO (black curve, full symbols) and Si${}_{3}$N${}_{4}$ (red curve, open symbols) substrate, all the unlabeled peaks belong to the substrates; (**b**) enlargement around the (110) peak.

The crystal structure of the as-deposited FePd/MgO and FePd/SiN samples is determined by the analysis of the spectra obtained by GIXRD and shown in Figure 2a. A body-centered cubic (BCC) structure is found in both cases. This evidence indicates the formation of a supersaturated solid solution of α-(Fe,Pd) as predicted by the FePd phase diagram and previously reported in the literature [47,48]. Considering the relative intensity of the diffraction peaks, no preferential orientation is observed. The used deposition parameters (see Section 2), such as a relatively high deposition rate and the room temperature of the substrates, lead to a polycrystalline structure of the growing thin film independently from the microstructure of the substrate. An enlargement of the spectra in the range 40–47° is shown in Figure 2b to compare the (110) peaks for the FePd film deposited on crystalline MgO (black line) and amorphous Si${}_{3}$N${}_{4}$ (red line) substrates. The peak is broader for the film deposited on the MgO substrate with respect to the corresponding one deposited on the Si${}_{3}$N${}_{4}$. In particular, the full width at half maximum (FWHM) value of the (110) peak is (2.56 ± 0.05)° and (1.63 ± 0.05)° for FePd/MgO and FePd/SiN sample, respectively. In a rough approximation, using the Scherrer formula [43], the average dimension (<*D*>) of the crystallites of the as-deposited FePd thin film can be estimated at about <*D*> = 33 and 52 nm for FePd/MgO and FePd/SiN sample, respectively. It is worth noting that the <*D*> values by Scherrer’s formula are generally underestimated [43] but, nevertheless, it can be said with confidence that the average size of the crystallites deposited on the amorphous Si${}_{3}$N${}_{4}$ substrate is greater if compared to the one of the FePd film deposited on the crystalline MgO substrate. This behavior provides evidence of the different structural features of the substrates which affect the crystalline grains dimension in the as-deposited FePd films. Therefore, the slight mismatch in the lattice parameter between the single crystalline MgO substrate (0.421 nm) [49] and the FePd thin film (0.376 nm) [50] provides substrate-induced stress/strain to the as-deposited film, resulting in the formation of grains with a smaller size. Conversely, the Si${}_{3}$N${}_{4}$ substrate with its amorphous texture does not provide any substrate-induced stress/strain to the film that grows effectively relaxed, leading to grains with a larger volume.

#### 3.1.2. Magnetic Properties

**Figure 3:**
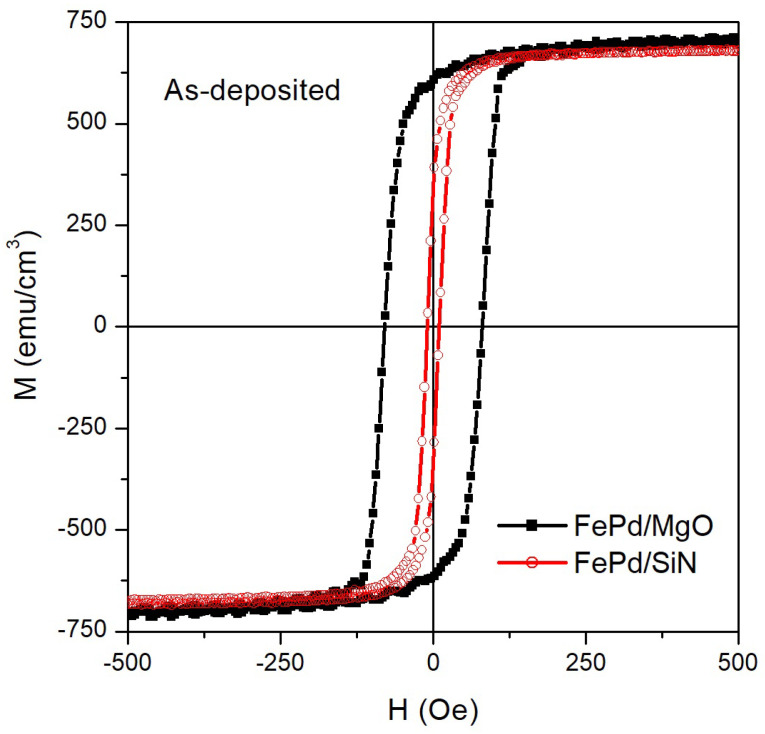
Room-temperature in-plane hysteresis loops of the as-deposited FePd samples on MgO (black curve) and Si${}_{3}$N${}_{4}$ (red curve) substrate.

Room-temperature in-plane hysteresis loops of the as-deposited FePd/MgO and FePd/SiN samples are shown in Figure 3. In both samples, the magnetization reversal occurs mainly for a single irreversible jump of the magnetization, indicating that the domain walls movement is the main mechanism governing the magnetization process. The magnetic susceptibility at the coercive field ($\chi_{Hc}$) is ≈2.7 × 10${}^{-3}$ Oe${}^{-1}$ and ≈4.2 × 10${}^{-2}$ Oe${}^{-1}$ for FePd/MgO and FePd/SiN, respectively.

The coercive field ($H_{c}$) and the normalized remanence ($M_{r}$/$M_{10kOe}$) of the as-deposited FePd thin film are influenced by the different structural properties of the underlying substrate. Both parameters reach higher values in the FePd/MgO sample with respect to the ones in the FePd/SiN sample: $H_{c}$ = 80 and 10 Oe; $M_{r}$/$M_{10kOe}$ = 0.84 and 0.47 for FePd/MgO and FePd/SiN, respectively. The difference in the $H_{c}$ values can be correlated to the average grain size of the as-deposited thin film [49]: the smaller grains in the FePd/MgO sample induce a large number of grain boundaries, crystal imperfections, and defects that hinder the motion of the domain walls and, consequently, increase the coercivity. On the other hand, the larger grains in the FePd/SiN make the magnetization reversal easier, leading to a lower coercive field value. The hindrance of the domain walls movement in the FePd/MgO sample, concurring to the increase in its $H_{c}$ value, also arises from the residual micro-stress [51] in the as-deposited FePd thin film induced by the slight mismatch of the lattice parameter and the one of the underlying substrate. Conversely, the amorphous texture of the Si${}_{3}$N${}_{4}$ substrate reduces the residual micro-stress, making the domain walls motion easier.

### 3.2. Dewetted Fe${}_{70}$Pd${}_{30}$ Thin Film

#### 3.2.1. Structural and Morphological Characterization

**Figure 4:**
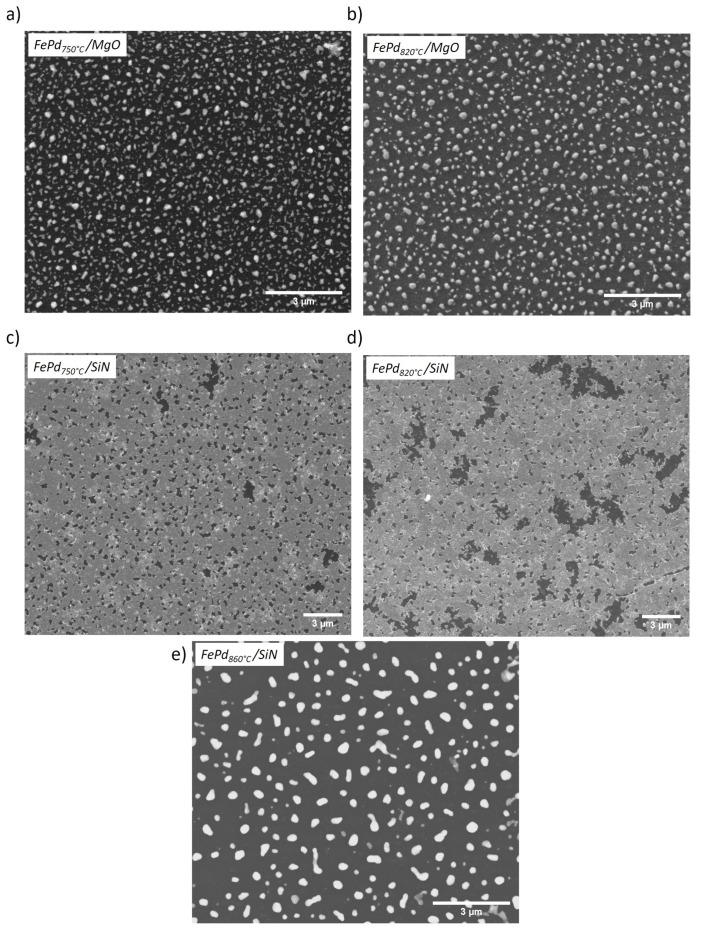
Morphology of Fe${}_{70}$Pd${}_{30}$ thin film deposited on MgO (panels **a**,**b**) and Si${}_{3}$N${}_{4}$ (panels **c**–**e**) substrate and annealed at selected temperature (see labels) for *t*${}_{A}$ = 55 min.

The thermally-assisted breakup of the highly flat continuous Fe${}_{70}$Pd${}_{30}$ layer by the solid-state-dewetting process is shown in Figure 4a,b and Figure 4c–e for the FePd/MgO and FePd/SiN samples, respectively. The high temperature values drive the morphological transformation by minimizing the free energy at the interface between the substrate and the thin film [18].

Clearly, the structural, compositional, and superficial features of the underlying substrate determine the kinetics of the solid-state-dewetting, the temperatures at which it starts and the different morphological properties of the final magnetic nanoparticles. The crystalline MgO substrate favors, already at *T*${}_{A}$ = 750 °C, the formation of well-separated magnetic nanoparticles (Figure 4a), although their irregular shape indicates that their interconnections have just separated. By increasing the annealing temperature up to *T*${}_{A}$= 820 °C, a more spherical-like shape of the NPs (Figure 4b) is developed by means of a process in which the free energy is further reduced and the system approaches equilibrium [52]. The exposure of the underlying MgO substrate is about 82% for both samples.

Conversely, annealing at the same temperature (*T*${}_{A}$ = 750 and 820 °C), the thermal energy provided to the as-deposited Fe${}_{70}$Pd${}_{30}$ layer on amorphous Si${}_{3}$N${}_{4}$ is not high enough to form separated magnetic nanoparticles. Indeed, the morphology of the FePd${}_{750^{\circ }C}$/SiN sample shows nucleated small empty space in the FePd film with irregular shape (i.e., holes that expose the underlying substrate) (Figure 4c) likely located at the grain boundaries of the as-deposited polycrystalline thin film [53,54]. Such formation of empty spaces in the continuous layer is the main feature that indicates the starting point of the solid-state dewetting process; in this case, the annealing parameters induce the exposure of the underlying Si${}_{3}$N${}_{4}$ substrate of about 12.8%. The increase in the annealing temperature up to *T*${}_{A}$ = 820 °C drives the spontaneous growth of the size of the holes (Figure 4d); consequently, in the FePd${}_{820^{\circ }C}$/SiN sample, the exposed area of the underlying substrate is increased up to 16.2%. Only a further increase in the thermal energy (*T*${}_{A}$ = 860 °C) provided to the as-deposited Fe${}_{70}$Pd${}_{30}$ thin film leads to the complete growth of the empty spaces with their consequent interconnection and the formation of well-separated FePd particles (Figure 4e). The exposure of the underlying Si${}_{3}$N${}_{4}$ substrate is, in this case, of about 83.4%.

**Figure 5:**
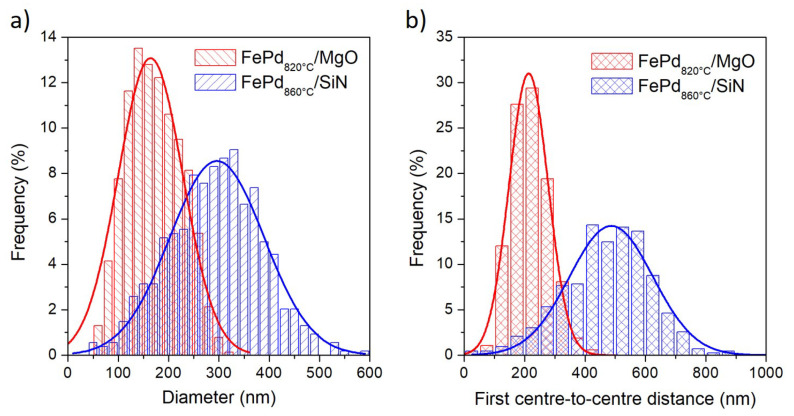
(**a**) Nanoparticles diameter distribution and (**b**) distribution of the center-to-center distance among first neighborhood of nanoparticles for the FePd${}_{820^{\circ }C}$/MgO and FePd${}_{860^{\circ }C}$/SiN samples. The Gaussian fits are plotted as full lines.

In order to compare the morphology obtained in FePd${}_{820^{\circ }C}$/MgO and FePd${}_{860^{\circ }C}$/SiN samples, a statistical analysis of the SEM images is performed. The distributions of the NPs diameter (<*D*>) and the center-to-center distance (<$d_{cc}$>) among the first neighborhood of nanoparticles are shown in Figure 5. The diameter distributions (Figure 5a) are fitted with a Gaussian curve with a mean value of <*D*> = 164 and 296 nm and a standard deviation of $\sigma_{D}$ = 128 and 187 nm for the FePd${}_{820^{\circ }C}$/MgO and FePd${}_{860^{\circ }C}$/SiN samples, respectively. Similarly, the distributions of the center-to-center distance (Figure 5b) are fitted with a Gaussian curve with a mean value of <$d_{cc}$> = 213 and 478 nm and a standard deviation of $\sigma_{cc}$ = 129 and 276 nm for the FePd${}_{820^{\circ }C}$/MgO and FePd${}_{860^{\circ }C}$/SiN samples, respectively. Consequently, the surface density (ρ) of NPs results in being much higher in the FePd${}_{820^{\circ }C}$/MgO (ρ ≈ 60 NPs/μm${}^{2}$) than in the FePd${}_{860^{\circ }C}$/SiN samples (ρ ≈ 15 NPs/μm${}^{2}$).

Therefore, the crystalline MgO substrate is observed to induce smaller (<*D*> reduced by a factor of ≈62%), closer (<$d_{cc}$> reduced by a factor of ≈55%), and more evenly distributed FePd magnetic NPs than the amorphous Si${}_{3}$N${}_{4}$ substrate.

**Figure 6:**
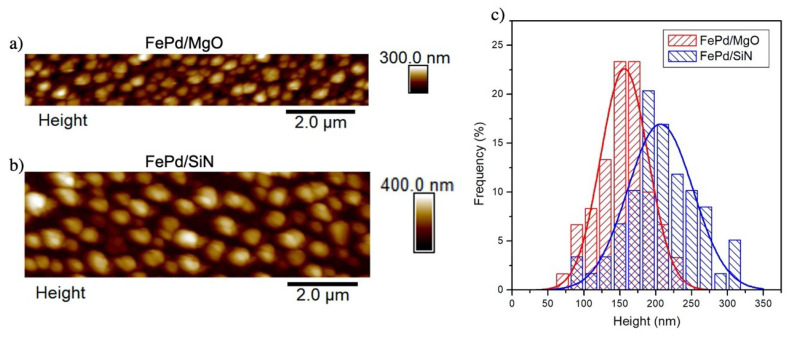
AFM images of dewetted samples: (**a**) FePd${}_{820^{\circ }C}$/MgO and (**b**) FePd${}_{860^{\circ }C}$/SiN samples; (**c**) nanoparticles height distribution with Gaussian fit (full line).

The height map of Fe${}_{70}$Pd${}_{30}$ nanoparticles lying on the MgO or Si${}_{3}$N${}_{4}$ substrate is obtained by the AFM technique; representative AFM images for the FePd${}_{820^{\circ }C}$/MgO and FePd${}_{860^{\circ }C}$/SiN samples are shown in Figure 6a,b. Statistical analysis of several AFM images allows to obtain the height distributions of the nanoparticles, which are shown in Figure 6c. Both distributions are roughly approximated by a Gaussian curve with a mean value of <*h*> = 156 and 206 nm and standard deviation of $\sigma_{h}$ = 65 and 91 nm for the FePd${}_{820^{\circ }C}$/MgO and FePd${}_{860^{\circ }C}$/SiN samples, respectively.

The estimation of the nanoparticles average aspect ratio (<*h*>/<*D*>) by combining the diameter and height information results in 0.95 ± 1.14 and 0.70 ± 0.75 for the FePd${}_{820^{\circ }C}$/MgO and FePd${}_{860^{\circ }C}$/SiN samples, respectively, revealing an almost spherical shape for the NPs obtained on the MgO substrate whereas a more oblate shape is revealed for the ones on the Si${}_{3}$N${}_{4}$ substrate.

These morphological evidences demonstrate that the substrate crystallinity influences the energy at the substrate/thin-film interface [23] as well as the thin-film structural properties (see Section 3.1). Consequently, the thermally activated atomic diffusion during the solid-state dewetting process can be controlled, leading to <*h*>/<*D*> and ρ of the dewetted FePd particles to meet the technological demands.

Moreover, the thermal energy provided to the thin films during the solid-state dewetting could lead to surface segregation effects of the bimetallic alloy [55], i.e., the enrichment of the surface in one of the components in comparison to the nominal concentration. In this context, the segregation of Au in Fe- and Ni-rich dewetted alloy is the subject of several studies [56,57,58]. In the case of FePd alloy, segregation of the Fe atoms on the surface was found, due to their higher oxide-forming tendency, compared to Pd atoms [59]. Therefore, a surface segregation effect in the dewetted samples studied in this work cannot be excluded a priori; however, further characterizations with more effective and in-depth techniques, such as X-ray photoemission spectroscopy (XPS) [60], atom probe [61] or atomic-resolution HAADF transmission microscopy [62] should be performed to have a more comprehensive picture.

#### 3.2.2. Magnetic Properties

**Figure 7:**
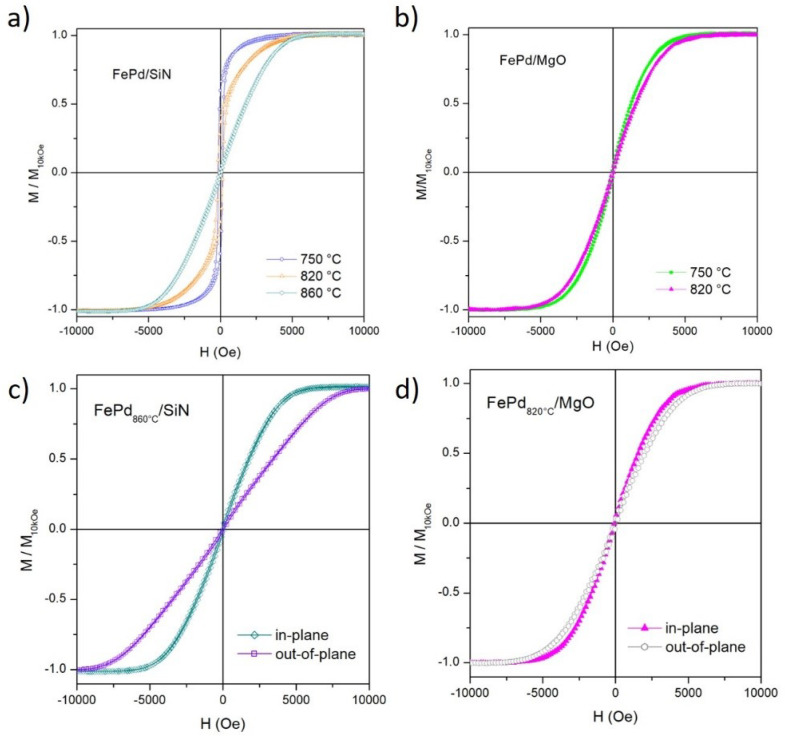
Room-temperature hysteresis loops: in-plane configuration for (**a**) FePd/SiN and (**b**) FePd/MgO samples as a function of the $T_{A}$; in-plane and out-of-plane magnetic hysteresis loops comparison for (**c**) FePd${}_{860^{\circ }C}$/SiN and (**d**) FePd${}_{820^{\circ }C}$/MgO.

Room-temperature in-plane hysteresis loops of the annealed samples are shown in Figure 7. All curves are normalized to the magnetic moment value measured at *H* = 10 kOe. The M(H) curves (Figure 7a,b) show a progressive and significant reduction in the magnetic susceptibility at the coercive field ($\chi_{Hc}$) as a function of $T_{A}$ (especially in the FePd/SiN samples; see Table 1) leading to a wider magnetic field interval in which the magnetic saturation is reached. This magnetic behavior indicates the appearance of a rotational mechanism of magnetization, which replaces the single irreversible reversal mechanism associated with the domain wall motion in the as-deposited thin film (see Figure 3 and discussion above) and, consequently, leads to a slower approach to saturation magnetization.

The measured magnetic properties are in strong correlation with the evolution of the Fe${}_{70}$Pd${}_{30}$ film morphology induced by the solid-state-dewetting process and observed in Figure 4. The remarkable increase of the $H_{c}$ value in the dewetted FePd${}_{750^{\circ }C}$/SiN and FePd${}_{820^{\circ }C}$/SiN samples (see Table 1), compared to the corresponding as-deposited thin film, is ascribed to the nucleation of the holes that act as pinning sites for the domain walls during the magnetization process of the whole sample. In particular, smaller holes (with a size smaller than the domain wall thickness) can directly pin the walls, whereas larger holes hinder the wall motion through the subsidiary domains around them, reducing the overall magnetostatic energy [51]. Instead, the rounder shape of the FePd well-separated nanoparticles, observed at the end of the solid-state-dewetting process in the FePd${}_{860^{\circ }C}$/SiN sample, induces a decrease in the effective magnetic anisotropy with a consequent reduction in the coercive field [25,63]. In this case, the magnetization process occurs independently in each nanoparticle mainly by rotational mechanisms: the magnetization vector rotates toward the applied field direction against the restoring force of the effective anisotropy, which includes a combination of the shape and the crystal anisotropy. As a consequence, a lower $\chi_{Hc}$ value is observed. Similar rotation mechanisms with comparable $\chi_{Hc}$ value are observed in the spherical shape magnetic NPs of the FePd${}_{750^{\circ }C}$/MgO and FePd${}_{820^{\circ }C}$/MgO samples (see Figure 4a,b). As expected, the improvement in the NPs roundness, induced increasing $T_{A}$ from 750 °C to 820 °C for FePd thin film on the MgO substrate, leads to a slight decrease in the $\chi_{Hc}$ and $H_{c}$ values (see Table 1). By comparing the coercive field values of the dewetted FePd${}_{820^{\circ }C}$/MgO and FePd${}_{860^{\circ }C}$/SiN samples, a multidomain configuration of the nanoparticles in both cases can be hypothesized since the decrease in the NPs average size <*D*> leads to an increase in the coercivity [51].

Room-temperature out-of-plane magnetic hysteresis loops of the FePd${}_{860^{\circ }C}$/SiN and FePd${}_{820^{\circ }C}$/MgO samples are measured and matched with the corresponding ones along the in-plane direction; see Figure 7c,d. In the FePd${}_{860^{\circ }C}$/SiN sample, the in-plane curve shows higher $\chi_{Hc}$ and lower saturation field, indicating that this direction is an anisotropy easy-axis of magnetization. Conversely, the in-plane and the out-of-plane hysteresis loops of the FePd${}_{820^{\circ }C}$/MgO sample appear almost perfectly superimposed. Such a features indicates a prevalent isotropic magnetic behavior with a random distribution in the space of the easy magnetization axis (only a very slight preference for the in-plane direction is still measurable). The area enclosed between the first branch of the in-plane hysteresis loop and the first branch of the out-of-plane one in the applied magnetic field interval 0–10 kOe is used to evaluate the effective anisotropy energy ($E_{eff}$) that the applied magnetic field spends to move the magnetization away from the easy-axis toward saturation along the hard axis [24,63]. As expected, the $E_{eff}$ value of the FePd8${}_{860^{\circ }C}$/SiN sample (≈1.1 × 10${}^{6}$ erg/cm${}^{3}$) is largely higher than that for FePd${}_{820^{\circ }C}$/SiN sample (2.6 × 10${}^{5}$ erg/cm${}^{3}$). These anisotropic results are in very good agreement with the morphological evidence reported and discussed in the previous section. The evident magnetic anisotropic behavior with the easy axis along the in-plane direction in FePd${}_{860^{\circ }C}$/SiN sample is excellently linked to the calculated oblate shape (<*h*>/<*D*> = 0.70 ± 0.75) of their FePd nanoparticles, whose major axis is in the plane of the film. Instead, the almost perfect spherical shape (<*h*>/<*D*> = 0.95 ± 0.14) of the nanoparticles in the FePd${}_{820^{\circ }C}$/MgO sample impact the magnetic isotropic behavior.

## 4. Conclusions

The proposed low-cost and “top-down” solid-state dewetting process is successfully used to nanostructure the as-deposited FePd thin film into an ordered nanoparticle array. The overall results indicate that the structural, compositional, and superficial features of the underlying substrate combined with the annealing parameters determine the kinetics of the solid-state-dewetting and, consequently, the final morphology, spatial arrangements and magnetic properties of the FePd nanoparticles.

The as-deposited Fe${}_{70}$Pd${}_{30}$ thin film with 30 nm thickness was successfully grown by the co-sputtering technique on both monocrystalline MgO and amorphous Si${}_{3}$N${}_{4}$ substrates. Morphological and structural characterizations reveal a flat, homogeneous, and continuous FePd layer, indicating a low strain accumulation at the substrate/film interface independently from the underlying substrate. On the other hand, the substrate affects the average size of the crystalline grains of the FePd thin film, resulting in bigger ones for the FePd/SiN, compared to the FePd/MgO. The magnetization reversal process is dominated in both as-deposited samples by the magnetic domain walls motion; the smaller grains size in the FePd/MgO sample results in a higher value of the coercive field.

The crystalline MgO substrate favors, by submitting the as-deposited FePd thin film to the dewetting process (annealing at $T_{A}$= 820 °C for $t_{A}$ = 55 min), the formation of well-separated magnetic nanoparticles with a spherical-like shape (<*D*> = 164 nm and <*h*> = 156 nm) and high surface density (ρ≈ 60 NPs/μm${}^{2}$). Conversely, exploiting the same annealing parameters, the layer deposited on the amorphous Si${}_{3}$N${}_{4}$ substrate leads to a hindering of the activation of the solid-state dewetting process leading only to the primary nucleation of the holes. An increase in the annealing temperature up to $T_{A}$ = 860 °C for $t_{A}$ = 55 min is required to complete propagation of the holes and to obtain the formation of well-separated FePd particles, which result in being higher, almost double in size, oblate in shape (<*D*> = 296 nm and <*h*> = 206 nm) and with a considerable lower surface density (ρ ≈ 15 NPs/μm${}^{2}$).

The spatial confinement of the magnetic materials induced by the solid-state dewetting remarkably affects the in-plane and out-of-plane magnetic properties. The magnetization reversal process in the magnetic FePd NPs array is mainly dominated by rotational mechanisms, leading to a slower approach to magnetic saturation with a significant reduction of the magnetic susceptibility at the coercive field, compared with the as-deposited thin film. The magnetization process occurs independently in each nanoparticle overcoming the shape and the crystal anisotropy. The coercive field is observed to increase as long as the holes and interconnection that act as pinning sites for the domain walls are still present in the sample. With the ending of the dewetting process, the spherical-like shape of the FePd nanoparticles leads to a reduction in the effective anisotropy with a consequent reduction in the coercive field. Such an increase in the coercivity as a function of the reduction in nanoparticle size (by comparing the FePd${}_{820^{\circ }C}$/MgO and FePd${}_{860^{\circ }C}$/SiN samples) is compatible with a multidomain configuration of the magnetization in each individual NP. Magnetic anisotropic features taken by the in-plane and out-of-plane hysteresis loops excellently support the average aspect ratio (<*h*>/<*D*>) values obtained by the morphological analysis: a slight magnetic anisotropic behavior is observed for the oblate shape of the nanoparticles in the FePd${}_{860^{\circ }C}$/SiN sample, whereas an almost perfect anisotropic behavior is in excellent agreement with the almost perfect spherical shape of the nanoparticles in the FePd${}_{820^{\circ }C}$/MgO sample.

In conclusion, the present structural, morphological, and magnetic characterizations prove that the substrate plays a primary role in the tuning of the dewetting process; therefore, a comprehensive structural analysis of the substrates and their interface with the magnetic layer will be one of the next crucial steps that allow to finely control the final morphology of magnetic FePd nanoparticles to meet the technological demands.

## Figures and Tables

**Table 1:** Annealing temperature ($T_{A}$), magnetic susceptibility at the coercive field ($\chi_{Hc}$), coercive field ($H_{c}$) for FePd/SiN and FePd/MgO samples.

	FePd/SiN		FePd/MgO	
$T_{A}$	$\chi_{\mathrm{Hc}}$	$H_{c}$	$\chi_{\mathrm{Hc}}$	$H_{c}$
750 °C	9.1 × 10${}^{-3}$	147	5.3 × 10${}^{-4}$	92
820 °C	4.2 × 10${}^{-3}$	177	4.1 × 10${}^{-4}$	88
860 °C	3.5 × 10${}^{-4}$	73	-	-

## Data Availability

The data presented in this study are available on request from the authors.
